# Induction of Activity-Regulated Cytoskeleton-Associated Protein and c-Fos Expression in an Animal Model of Anorexia Nervosa

**DOI:** 10.3390/nu15173830

**Published:** 2023-09-01

**Authors:** Maria Scherma, Maria Francesca Palmas, Augusta Pisanu, Paolo Masia, Simona Dedoni, Chiara Camoglio, Walter Fratta, Anna R. Carta, Paola Fadda

**Affiliations:** 1 Division of Neuroscience and Clinical Pharmacology, Department of Biomedical Sciences, University of Cagliari, 09042 Cagliari, Italy; mariaf.palmas@unica.it (M.F.P.); dedoni@unica.it (S.D.); chiara.camoglio@unica.it (C.C.); wfratta@unica.it (W.F.); acarta@unica.it (A.R.C.); pfadda@unica.it (P.F.); 2 Neuroscience Institute, Section of Cagliari, National Research Council (CNR), 09042 Cagliari, Italy; augusta.pisanu@in.cnr.it

**Keywords:** anorexia nervosa, activity-regulated cytoskeleton-associated protein, prefrontal cortex, nucleus accumbens, hippocampus

## Abstract

Anorexia nervosa (AN) is a complex eating disorder characterized by reduced caloric intake to achieve body-weight loss. Furthermore, over-exercise is commonly reported. In recent years, animal models of AN have provided evidence for neuroplasticity changes in specific brain areas of the mesocorticolimbic circuit, which controls a multitude of functions including reward, emotion, motivation, and cognition. The activity-regulated cytoskeleton-associated protein (Arc) is an immediate early gene that modulates several forms of synaptic plasticity and has been linked to neuropsychiatric illness. Since the role of Arc in AN has never been investigated, in this study we evaluated whether the anorexic-like phenotype reproduced by the activity-based anorexia (ABA) model may impact its expression in selected brain regions that belong to the mesocorticolimbic circuit (i.e., prefrontal cortex, nucleus accumbens, and hippocampus). The marker of neuronal activation c-Fos was also assessed. We found that the expression of both markers increased in all the analyzed brain areas of ABA rats in comparison to the control groups. Moreover, a negative correlation between the density of Arc-positive cells and body-weight loss was found. Together, our findings suggest the importance of Arc and neuroplasticity changes within the brain circuits involved in dysfunctional behaviors associated with AN.

## 1. Introduction

Anorexia nervosa (AN) is a complex eating disorder that affects predominantly females and ranks among the highest death rate of any mental disease [1]. Lower normal body weight, along with an intense fear of gaining weight, are two key features of AN [2]. In addition, high physical activity is frequently reported to optimize weight loss and is considered one of the primary factors in the maintenance of AN [3]. Finally, AN often coexists with other psychiatric disorders, including depression and/or anxiety [4]. The etiology underlying AN is not fully elucidated and specific knowledge is required to better understand the neural correlates driving this disorder. In recent years, animal models of AN have provided evidence for neuroplasticity changes in specific brain areas of the mesocorticolimbic circuit, which could contribute to the progression of this disorder [5]. This circuitry, which consists of projections from the ventral tegmental area to the nucleus accumbens and other limbic-related regions, including the hippocampus and prefrontal cortex, controls a multitude of functions that are relevant to AN, including reward, emotion, motivation, and cognition, as well as homeostatic and hedonic aspects of feeding behavior [6]. Using the “activity-based anorexia” (ABA) model, which mimes important key symptoms of the human condition (i.e., severe food restriction, weight loss, and hyperactivity), atypical dendritic arborization of hippocampal CA1 neurons was found in ABA rats compared to controls [7,8]. Moreover, ABA induction seems to cause a redistribution of N-methyl-D-aspartate (NMDA) receptors in the hippocampus, predominantly among ABA rats showing severe weight loss [9]. Consistent with impaired glutamatergic signaling, ABA rats showed alterations in the structure and composition of the glutamatergic synapse in both the nucleus accumbens and the medial prefrontal cortex [10,11]. Finally, the expression levels of brain-derived neurotrophic factor (BDNF), an activity-dependent modulator of neuroplasticity, have been found to be altered in specific regions of the mesocorticolimbic pathway of ABA rats, including the medial prefrontal cortex and the amygdala [12,13]. Activity-regulated cytoskeleton-associated protein (Arc) is a brain-enriched immediate early gene (IEG) linked to multiple forms of synaptic plasticity including modification of synapse structure and strength [14] (Shepherd et al. 2011). Under basal conditions, Arc expression is low, but it is strongly induced in response to synaptic activation in different brain structures [15]. Moreover, Arc expression is increased by BDNF [16]. Changes in Arc expression has been linked to psychiatric conditions, however, its role in AN has never been investigated. Hence, in this study we evaluated whether the typical anorexic-like phenotype reproduced by the ABA model, may impact Arc expression in selected brain regions of the mesocorticolimbic pathway (i.e., prefrontal cortex, nucleus accumbens, and hippocampus). We also assessed neuronal activation by measuring the expression of c-Fos in the same brain areas. Finally, we investigated any relationships between wheel-running activity and body weight changes induced by the ABA protocol, and the observed molecular alterations.

## 2. Material and Methods

### 2.1. Animals and Housing

Subjects were 32 adolescent female Sprague Dawley rats (Envigo, Italy) weighing 125–150 g at the start of the study (post-natal day: ~42–49). Upon arrival, animals were housed, four per cage, in a climate-controlled animal room (21 ± 2 °C; 60% humidity) under a reversed 12-h light/12-h dark cycle (lights on at 12:00 a.m. and fed standard rat chow and water ad libitum. Animal care and experimental procedures were conducted in accordance with the Italian (D.L. 26/2014) and European Council directives (63/2010) and were approved by the Ethical Committee for Animal Experiments at the University of Cagliari (Sardinia, Italy) and the Italian Department of Health (287/2016). Every possible effort was made to minimize the pain and discomfort of the animals, and to reduce the number of experimental subjects. ABA rats were not allowed to lose more than 25% of their initial body weight [17].

### 2.2. Experimental Design

**Figure 1:**
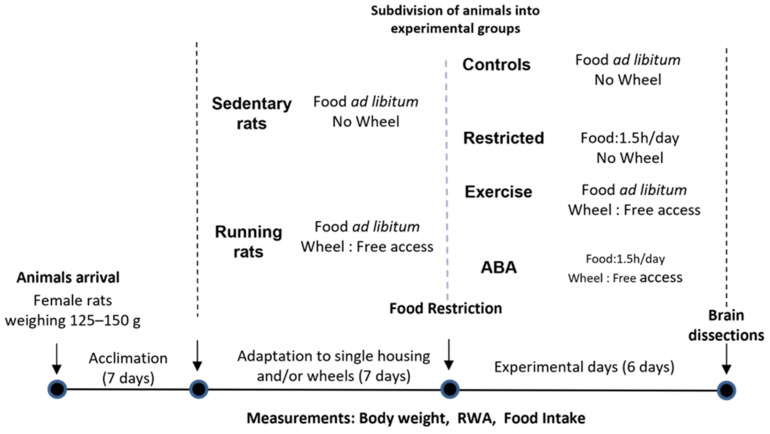
Experimental design.

Figure 1 show a schematic representation of the experimental design. Apparatus and procedure were the same as previously described [17,18]. In summary, after 1 week of ac-climatization, rats were housed singly and randomly divided into: sedentary rats (*n* = 16 rats per group, housed in standard polycarbonate cages) and running rats (*n* = 16 rats per group, housed in polycarbonate cages equipped with running wheels (Ugo Basile Activity wheel, Ugo Basile, Varese, Italy)). Animals were adapted to their housing conditions for 7 days with ad libitum food and running wheel access (where applicable). Every day, 30 min before the shift to the dark cycle, body weight, 24 h food intake and RWA were monitored. At the end of the adaptation phase, rats from each group (sedentary and running rats) were randomly separated into two cohorts of animals (*n* = 8 rats per group) according to their protocol conditions: (1) ‘Control’: sedentary + 24 h/food access; (2) ‘Restricted’: sedentary + 1.5 h/food access; (3) ‘Exercise’: activity wheel + 24 h/food access; (4) ‘ABA’: activity wheel + 1.5 h/food access. 1.5 h after the start of the 12 h dark cycle, food was completely removed from the cages of ABA and Restricted groups. Throughout the food restriction period (6 days) a pre-weighed amount of food was provided for 1.5 h each day, at the beginning of the dark phase. At the end of this free feeding period, remaining food was removed and weighted to measure food consumption. During the remaining 22.5 h, ABA group had free access to the wheel. Exercise and Control groups continued to have food ad libitum, and Exercise rats had free access to the wheel. 30 min before the shift to the dark cycle, daily body weight, 24 h food intake (Control and Exercise groups) and RWA (Exercise and ABA groups) were monitored.

### 2.3. Immunohistochemical Procedures

At the end of the 12 h light phase on day 6 of the ABA induction phase, rats were deeply anesthetized (chloral hydrate, 500 mg/kg, i.p., 2 mL/kg) and perfused transcardially in ice-cold 0.1 M PBS (pH 7.4), followed by 4% paraformaldehyde. Thereafter, their brains were post-fixed overnight in 4% paraformaldehyde and stored in 0.1% NaN_3_-PBS at 4 °C. Free-floating coronal brain sections of 40 μm thickness were vibratome-cut at the level of the prelimbic and infralimbic prefrontal cortex (Bregma: +3.72 to +2.52), nucleus accumbens shell and core (Bregma: +2.28 to +1.08), dorsal hippocampus (Bregma: −2.16 to −4.08), and ventral hippocampus (Bregma: −5.16 to −6.12), according to the atlas of Paxinos and Watson [19]. For Arc and c-Fos quantification, sections were pre-incubated in BSA/normal donkey serum blocking solution and then immunoreacted with mouse monoclonal Arc (C-7) (1:1000, Santa Cruz Biotechnology, Santa Cruz, CA, USA; sc-17839) and sheep polyclonal c-Fos (1:2000, Sigma Aldrich, St Louis, MO, USA; AB1584) primary antibodies. The reaction was then amplified using the proper biotinylated secondary antibody and visualized by the classic avidin–peroxidase complex (ABC, Vector Laboratories, Burlingame, CA USA; VEC.PK-6100) protocol, using 3,30-diaminobenzidine (Sigma-Aldrich, St. Louis, MO, USA; D4418) as a chromogen.

### 2.4. Image Acquisition and Analysis of c-Fos- and Arc-Positive Elements

Images were acquired with a Zeiss AxioScopeA1 microscope (Carl Zeiss, Jena, Germany) connected to a digital camera (1.4 MegaPixels, Infinity 3–1, Lumenera Corporation, Ottawa, ON, Canada) with 10X magnification. The c-Fos and Arc immunoreactivity (Arc) across different brain regions were quantified by manually selecting non-overlapping regions of interest (ROIs) of the four brain regions. All analyses were performed and analyzed by an experimenter blinded to the experimental conditions. The numbers of c-Fos- and Arc-positive cells were then counted within each ROI, applying the “entropy threshold” and “analyze particles” tools of ImageJ software (National Institutes of Health, Bethesda, MD, USA), with parameters for positive particles properly set at a range of 30–130 pixels for particle size and 0.6–1.0 for particle circularity, as previously described [20]. Slices that were excessively damaged, such as cutting-damaged tissue within the regions of interest, were excluded before staining. About 5% of the total sections/ROIs, exhibiting tissue or staining artefacts that may interfere with automated analysis, such as cuts, DAB overstaining, or excessive spots of dirt, were excluded [20].

### 2.5. Statistical Analysis

Body weight, food intake, and RWA are presented as the mean ± SEM and were analyzed by two-way ANOVA for repeated measures. Data on Arc and c-Fos were expressed as the average of Arc- or c-Fos-positive cells/μm^2^ in three different sections of each region of interest. We analyzed differences between groups using a one-way ANOVA. Post hoc comparisons were made using Bonferroni multiple comparisons test or Tukey’s multiple comparisons test where appropriate. Pearson’s correlation analysis was used to test associations between behavioral outcomes induced by the ABA procedure and molecular changes. Analysis of data was carried out using Prism 8.2.1 (GraphPad Software, Prism v8.2.1, San Diego, CA, USA). In all cases, differences with *p* < 0.05 were considered significant.

## 3. Results

### 3.1. ABA Induction

**Figure 2:**
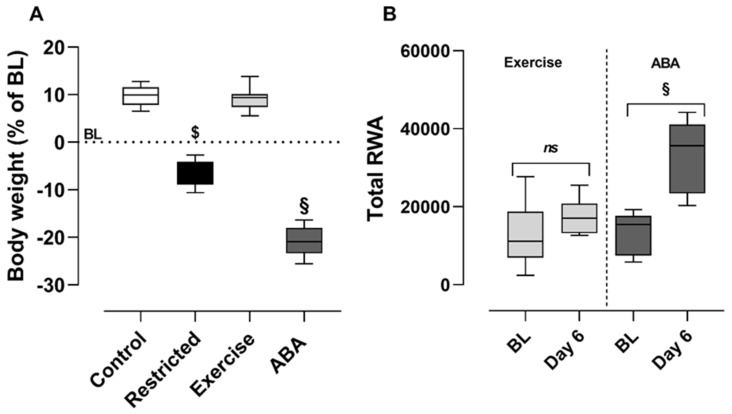
(**A**) Body weight and (**B**) RWA in the Control, Exercise, Restricted, and ABA groups at the end of the ABA induction (day 6): Results are presented as the mean ± SEM (*n* = 8 rats per group). Body weight: statistical analysis was performed by one-way ANOVA, followed by Bonferroni’s post hoc test, § *p* < 0.001 ABA vs. Control, Exercise, and Restricted; $ *p* < 0.001 Restricted vs. Control, and Exercise; RWA: statistical analysis was performed by a paired Student’s *t*-test; ABA § *p* < 0.001 BL vs. Day 6.

In line with our previous studies, the ABA and Restricted groups exhibited significant body-weight loss, while the Control and Exercise groups gained weight (Table 1) [17,18]. Two-way ANOVA revealed a significant main effect of group × time interaction (F (18,168) = 81.14, *p* < 0.05). On day 6, the percentage of body-weight loss in the ABA group was significantly more pronounced than that in the Restricted group (−20.81% ± 1.078 and −7.14% ± 0.99 from BL, respectively), while no differences in body weight were found between Control and Exercise groups (+ 9.75% ± 0.77 and + 9.176% ± 0.99 from BL, respectively) (Figure 2A). Due to the limited access, daily food intake in both the ABA and Restricted groups was lower than that of rats fed *ad libitum* (the Control and Exercise groups), and two-way ANOVA revealed a significant main effect of group × time interaction (F (15,140) = 10.12, *p* < 0.05; Table 1). We also confirmed that the ABA rats showed significantly higher RWA than the Exercise rats (Table 1). Two-way ANOVA revealed a significant group × time interaction effect (F (6, 42) = 7.096, *p* < 0.05). On day 6, the ABA rats displayed a 146% increase in RWA relative to the BL (paired *t*-test: *t* (7) = 10.25, *p* < 0.0001), while Exercise rats only showed a 31% increase (paired *t* -test: *t* (7) = 2.100, *p* = 0.0728, +31%) (Figure 2B).

### 3.2. Effect of ABA Induction on the Expression of Arc and c-Fos

#### 3.2.1. Hippocampus

We analyzed both hippocampal subdivisions (i.e., dorsal and ventral) and their subregions (CA1, CA3, and the dentate gyrus).

##### Dorsal Hippocampus

**Figure 3:**
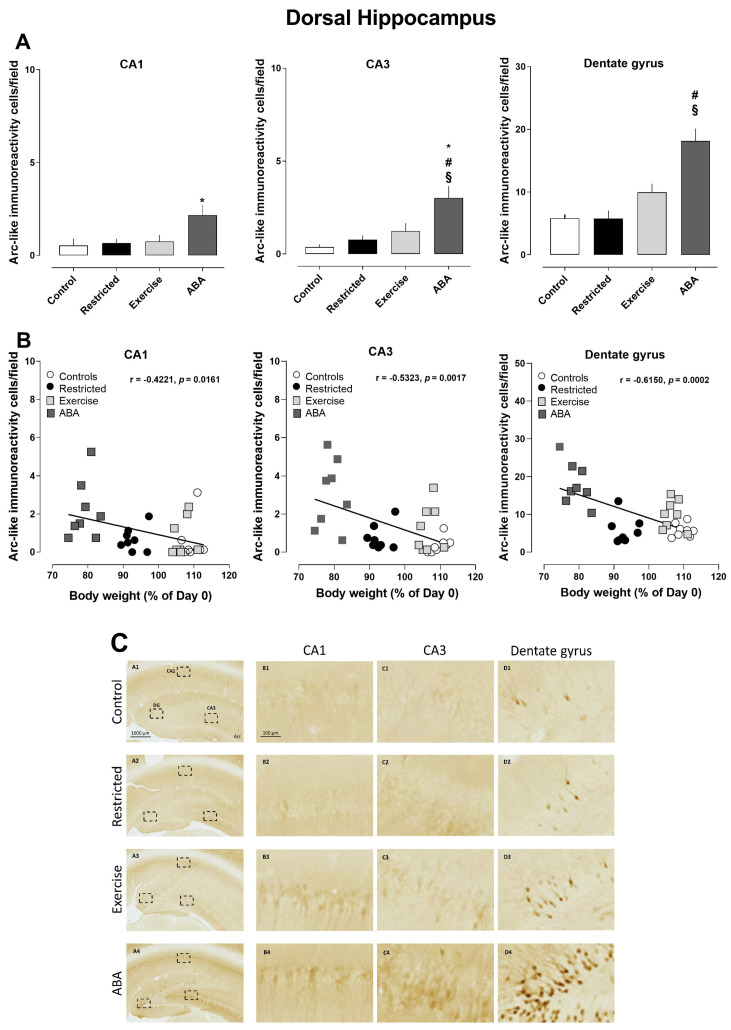
Arc expression in the dorsal hippocampus, and its correlation with body weight (%): (**A**) Stacked bar charts show the counts of Arc-positive nuclei/field in the CA1, CA3, and dentate gyrus subfields. Values are expressed as the mean ± SEM of positive nuclei/field (one-way ANOVA followed by Tukey’s post hoc test). CA1: * *p* < 0.05 ABA vs. Control; CA3: * *p* < 0.05 ABA vs. Exercise, # *p* < 0.01 ABA vs. Restricted, and § *p* < 0.0001 ABA vs. Control; dentate gyrus: # *p* < 0.01 ABA vs. Exercise and § *p* < 0.0001 ABA vs. Control and Restricted. (**B**) Correlation between Arc expression and body weight in Control, Restricted, Exercise, and ABA. (**C**) Representative images of Arc-like immunoreactive elements in the dorsal hippocampus. (A–D) Column A shows regional distribution of Arc-like immunoreactive elements in representative dorsal hippocampi of Control (row 1), Restricted (row 2), Exercise (row 3), and ABA (row 4) rats. Columns B, C, and D show higher magnifications of Arc immunoreactivity of (B) CA1, (C) CA3, and (D) the dentate gyrus in the regions of interest (rectangles) represented in column A. Scale bars: 1000 µm; 100 µm.

At the end of the ABA induction, the density of Arc-positive cells significantly increased in the dorsal hippocampus of the ABA group, compared to what was observed in the other experimental groups (one-way ANOVA: CA1 (F (3, 28) = 3.872, *p* = 0.0196; CA3 (F (3, 28) = 8.169, *p* = 0.0005; dentate gyrus (F (3, 28) = 17.51, *p* < 0.0001) (Figure 3A). Pearson’s correlation analyses revealed that the density of Arc-positive cells was negatively correlated with body weight (CA1 r = −0.4221, *p* = 0.0161; CA3 r = −0.5323, *p* = 0.0017; dentate gyrus r = −0.6150, *p* = 0.0002) (Figure 3B), but not with RWA.

**Figure 4:**
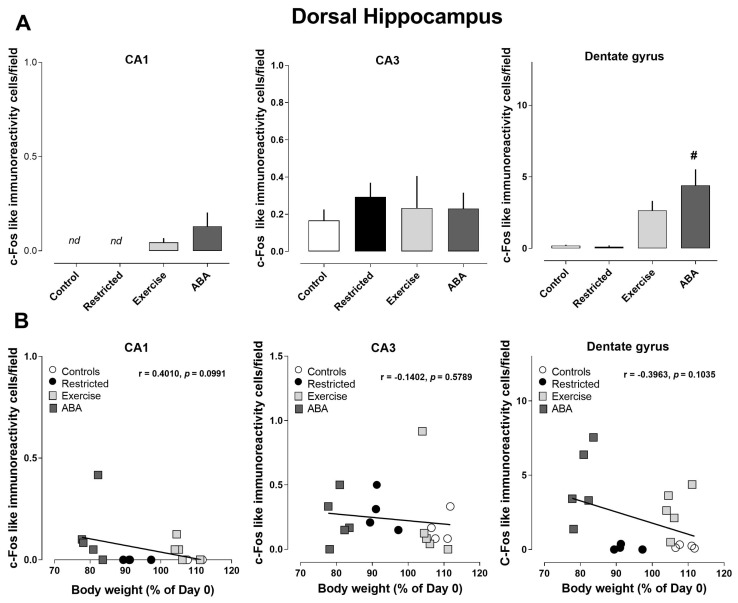
Expression of c-Fos in the dorsal hippocampus, and its correlation with body weight (%): (**A**) Stacked bar charts show the counts of c-Fos-positive nuclei/field in the CA1, CA3, and dentate gyrus subfields of Control, Restricted, Exercise, and ABA rats. Values are expressed as the mean ± SEM of positive nuclei/field (one-way ANOVA followed by Tukey’s post hoc test). CA3: # *p* < 0.01 ABA vs. Control and Restricted. (**B**) Correlation between Arc expression and body weight in Control, Restricted, Exercise, and ABA rats.

As regards c-Fos, the only significant change was detected in the dentate gyrus of ABA rats compared to the other experimental groups (one-way ANOVA: (F (3, 14) = 7.946, *p* = 0.0025) (Figure 4A). No correlation was found with body weight (CA1 r = −0.4010, *p* = 0.0991; CA3 r = −0.1402, *p* = 0.5789; dentate gyrus r = −0.3963, *p* = 0.1035) (Figure 4B).

##### Ventral Hippocampus

**Figure 5:**
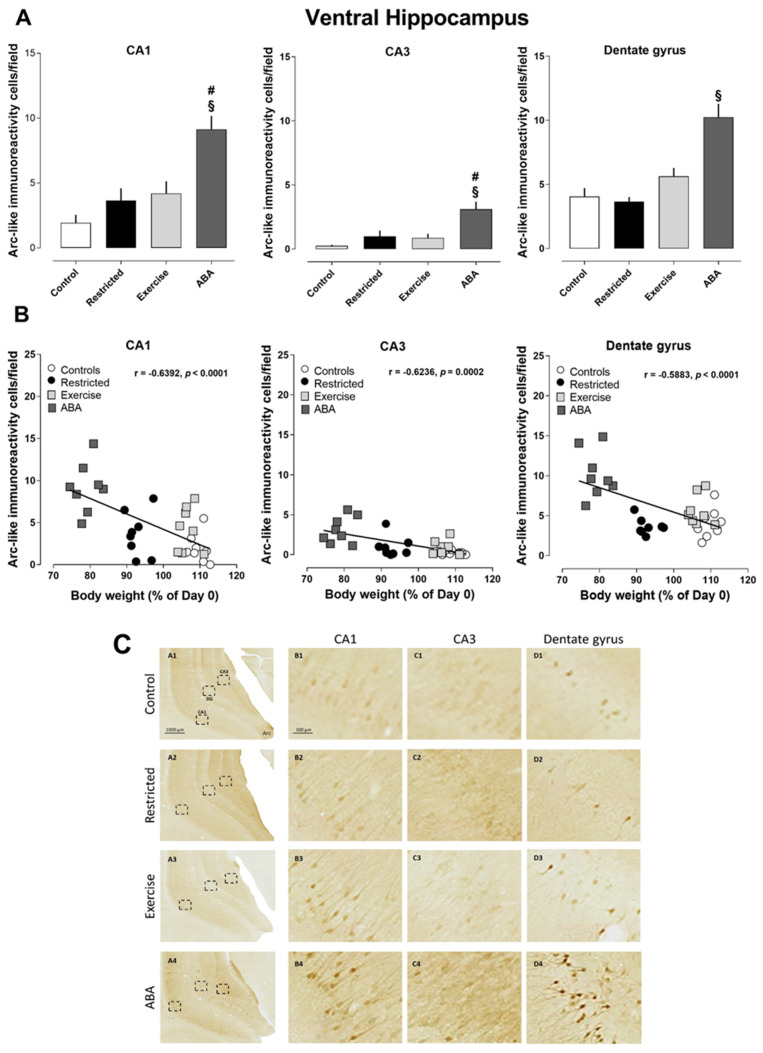
Arc expression in the ventral hippocampus, and its correlation with body weight (%): (**A**) Stacked bar charts show the counts of Arc-positive nuclei/field in the CA1, CA3, and dentate gyrus subfields. Values are expressed as the mean ± SEM of positive nuclei/field (one-way ANOVA followed by Tukey’s post hoc test). CA1: # *p* < 0.01 ABA vs. Restricted and Exercise, § *p* < 0.0001 ABA vs. Control; CA3: # *p* < 0.01 ABA vs. Restricted and Exercise, § *p* < 0.0001 ABA vs. Control; dentate gyrus: § *p* < 0.0001 ABA vs. Control, Restricted, and Exercise. (**B**) Correlation between Arc expression and body weight in Control, Restricted, Exercise, and ABA rats. (**C**) Representative images of Arc-like immunoreactive elements in the ventral hippocampus. (A–D) Column A shows the regional distribution of Arc-like immunoreactive elements in representative ventral hippocampi of Control (row 1), Restricted (row 2), Exercise (row 3), and ABA (row 4) rats. Columns B, C, and D show higher magnifications of Arc immunoreactivity of (B) CA1, (C) CA3, and (D) the dentate gyrus in the regions of interest (rectangles) represented in column A. Scale bars: 1000 µm; 100 µm.

**Figure 6:**
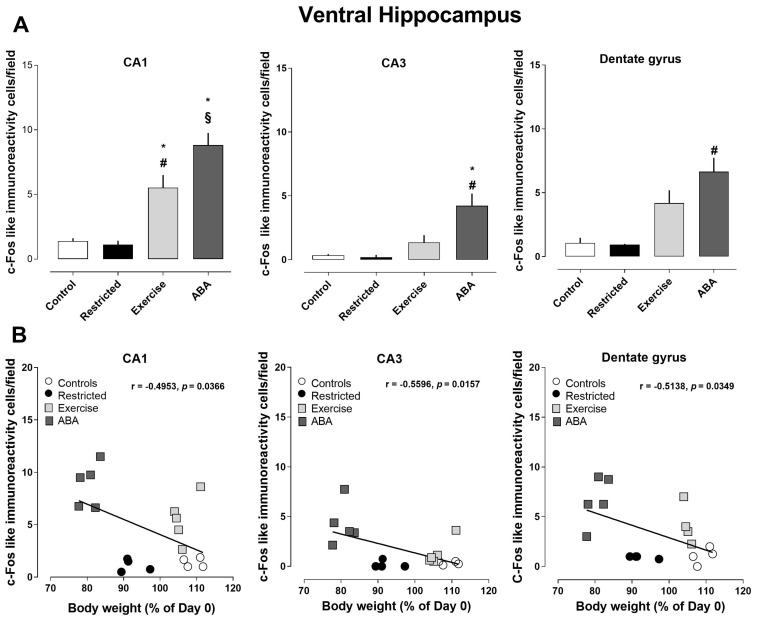
Expression of c-Fos in the ventral hippocampus, and its correlation with body weight (%): (**A**) Stacked bar charts show the counts of c-Fos-positive nuclei/field in the CA1, CA3, and dentate gyrus subfields of Control, Restricted, Exercise, and ABA rats. Values are expressed as the mean ± SEM of positive nuclei/field (one-way ANOVA followed by Tukey’s post hoc test). CA1: * *p* < 0.05 ABA vs. Exercise and § *p* < 0.0001 ABA vs. Control and Restricted, * *p* < 0.05 Exercise vs. Control and # *p* < 0.001 vs. Restricted; CA3: * *p* < 0.05 ABA vs. Exercise and # *p* < 0.001 ABA vs. Control and Restricted; dentate gyrus: # *p* < 0.001 ABA vs. Control and Restricted. (**B**) Correlation between Arc expression and body weight in Control, Restricted, Exercise, and ABA rats.

Similarly, to what was seen in the dorsal hippocampus, the density of Arc-positive cells significantly increased in all subregions of the ventral hippocampus of ABA rats (one-way ANOVA: CA1 F (3, 28) = 3.872, *p* < 0.0001; CA3 (F (3, 28) = 9.430, *p* = 0.0002; dentate gyrus (F (3, 28) = 17.40, *p* < 0.0001) (Figure 5A). Pearson’s correlation analyses revealed that the density of Arc-positive cells was negatively correlated with body weight (CA1 r = −0.6392, *p* < 0.0001; CA3 r = −0.6236, *p* < 0.0001; dentate gyrus r = −0.5883, *p* = 0.0004) (Figure 5B). Moreover, c-Fos immunoreactivity was found to be increased in all three analyzed subregions of the ventral hippocampus of the ABA group, as well as in the CA1 of the Exercise group when compared to the other experimental groups (one-way ANOVA: CA1 F (3, 14) = 21.97, *p* < 0.0001; CA3 F (3, 14) = 9.022, *p* = 0.0002; DG F (3, 13) = 11.61, *p* = 0.0060) (Figure 6A). As shown in Figure 6B, this effect was negatively correlated with body weight (CA1 r = −0.4953, *p* = 0.0366; CA3 r = −0.5596, *p* = 0.0157; dentate gyrus r = −0.5138, *p* = 0.0014) (Figure 6B). However, no correlation was found with RWA.

#### 3.2.2. Nucleus Accumbens

**Figure 7:**
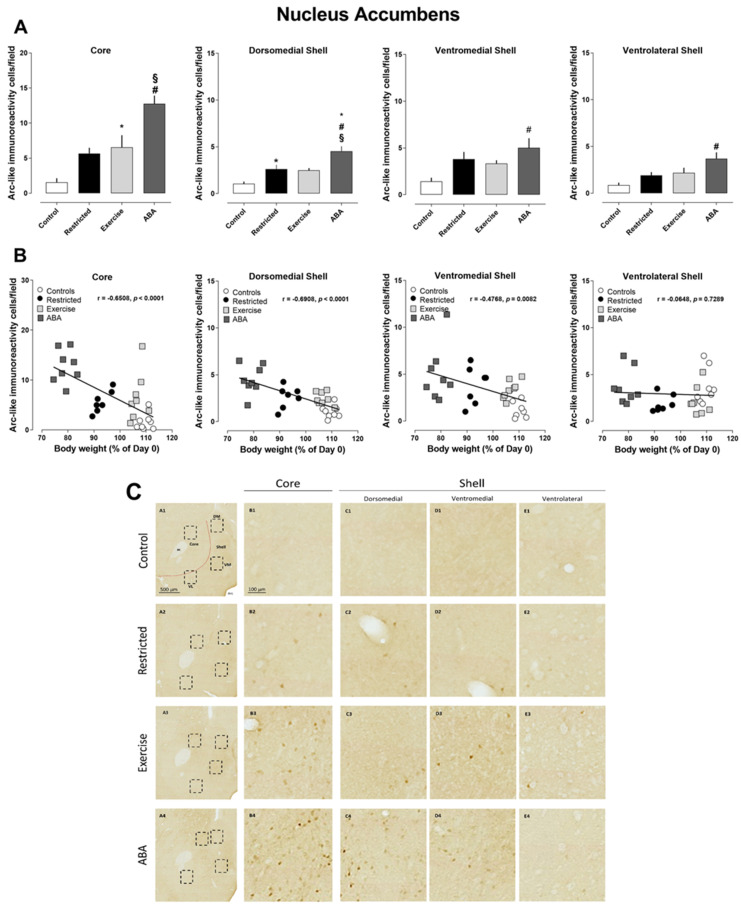
Arc expression in the nucleus accumbens, and its correlation with body weight (%): (**A**) Stacked bar charts show the counts of Arc-positive nuclei/field in the dorsomedial, ventromedial, and ventrolateral shells of the nucleus accumbens. Values are the mean ± SEM of positive nuclei/field (one-way ANOVA followed by Tukey’s post hoc test). Core: § *p* < 0.0001 ABA vs. Control, # *p* < 0.01 ABA vs. Restricted and Exercise; * *p* <0.05 Exercise vs. Control. Dorsomedial shell: * *p* < 0.05 ABA vs. Restricted, # *p* < 0.01 ABA vs. Exercise, § *p* < 0.0001 ABA vs. Control; * *p* < 0.05 Restricted vs. Control. Ventromedial and ventrolateral shells: #*p* < 0.01 ABA vs. Control. (**B**) Correlation between Arc expression and body weight in Control, Restricted, Exercise, and ABA rats. (**C**) Representative images of Arc-like immunoreactive elements in the nucleus accumbens. (A–E) Column A shows the regional distribution of Arc-like immunoreactive elements in representative nuclei accumbens of Control (row 1), Restricted (row 2), Exercise (row 3), and ABA (row 4) rats. Columns B, C, and E show higher magnifications of Arc immunoreactivity of the (B) core, (C) dorsomedial shell, (D) ventromedial shell, and (E) ventrolateral shell in the regions of interest (rectangles) represented in column A. Scale bars: 500 µm; 100 µm. Dashed red lines mark the boundaries of core and shell subregions.

**Figure 8:**
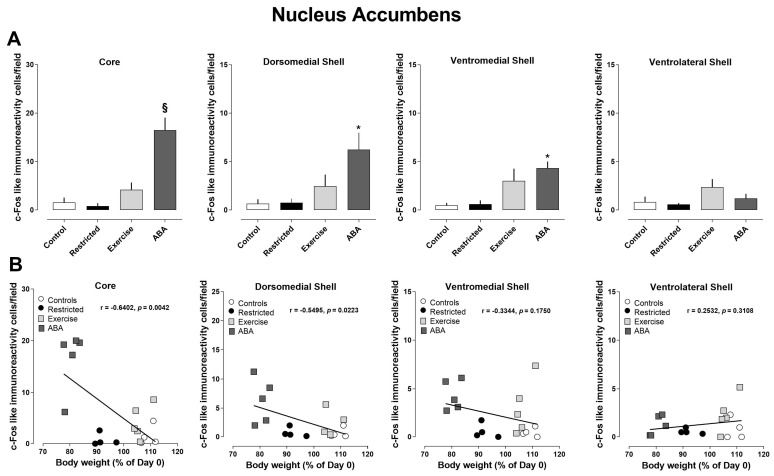
Expression of c-Fos in the nucleus accumbens, and its correlation with body weight (%): (**A**) Stacked bar charts show the counts of c-Fos-positive nuclei/field in the core and the dorsomedial, ventromedial, and ventrolateral shells of the nucleus accumbens. Values are the mean ± SEM of positive nuclei/field (one-way ANOVA followed by Tukey’s post hoc test). Core: § *p* < 0.001 ABA vs. Control, Restricted, and Exercise; dorsomedial and ventromedial shells: * *p* < 0.05 ABA vs. Control and Restricted. (**B**) Correlation between Arc expression and body weight in Control, Restricted, Exercise, and ABA rats.

As shown in Figure 7A, the density of Arc-positive cells significantly increased in the nucleus accumbens of the ABA group. More specifically, the increase was found in all analyzed subregions of the nucleus accumbens compared to those of the other experimental groups (one-way ANOVA: core, F (3, 27) = 16.06, *p* = 0.0374; dorsomedial shell, F (3, 27) = 13.85, *p* < 0.0001; ventromedial shell, F (3, 27) = 4.811, *p* = 0.0082; ventrolateral shell, F (3, 27) = 5.835, *p* = 0.0033). Moreover, post hoc analysis revealed that the density of Arc-positive cells was also found to be significantly increased in the core of the Exercise group, as well as in the dorsomedial shell of the Restricted group. Again, we observed a significant inverse correlation between the density of Arc-positive cells and body weight in all subregions analyzed, except in the ventrolateral shell (core r = −0.6508, *p* < 0.0001; dorsomedial shell r = −0.6908, *p* < 0.0001; ventromedial shell r = −0.4768, *p* = 0.0067; ventrolateral shell r = 0.0648, *p* = 0.7289) (Figure 7B). The c-Fos immunoreactivity was found to be increased only in the in the core, as well as in the dorsomedial shell of the ABA group (Figure 8A)—an effect that was negatively correlated with body weight (Figure 8B) (core, r = −0.6402, *p* = 0.0042; dorsomedial shell, r = −0.5495, *p* = 0.0223; ventromedial shell, r = −0.3344, *p* = 0.1750; ventrolateral shell, r = 0.2532, *p* = 0.3300), but not with RWA.

#### 3.2.3. Prefrontal Cortex

**Figure 9:**
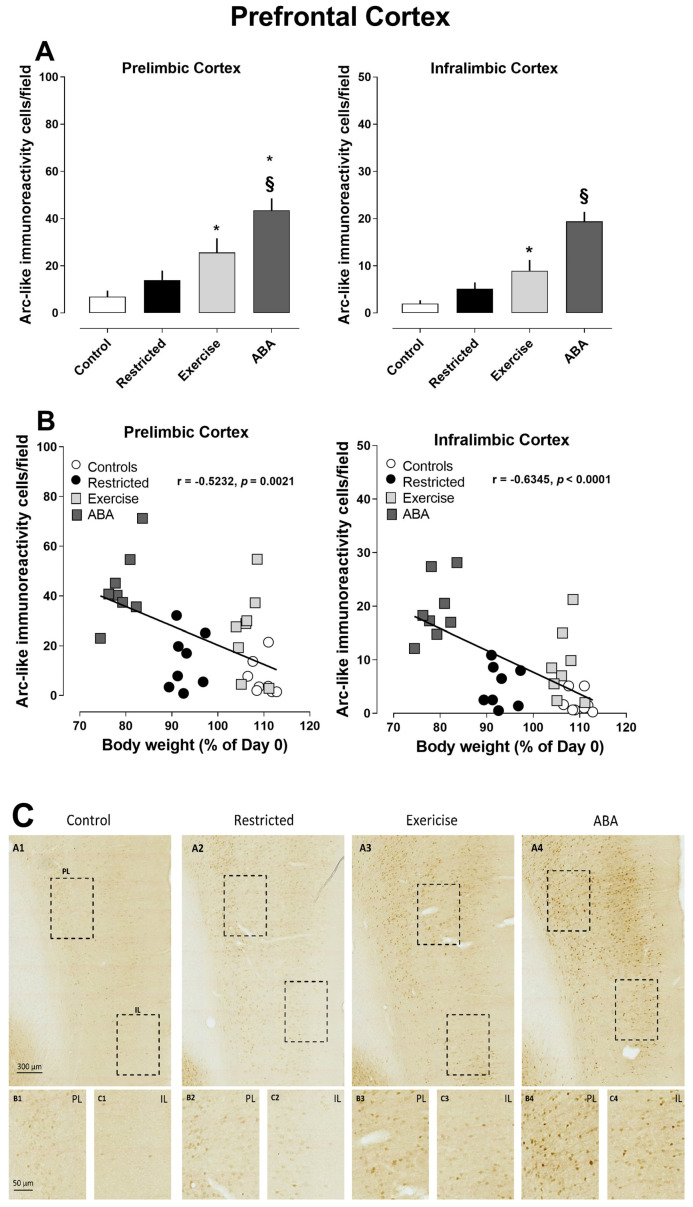
Arc expression in the prefrontal cortex, and its correlation with body weight (%): (**A**) Stacked bar charts show the counts of Arc-positive nuclei/field in the prefrontal cortex. Values represent the mean ± SEM of positive nuclei/field and (one-way ANOVA followed by Tukey’s post hoc test). Prelimbic Cortex: * *p* < 0.05 ABA vs Exercise, § *p* < 0.0001 ABA vs Control and Restricted; * *p* < 0.05 Exercise vs Control. Infralimbic Cortex: § *p* < 0.0001 ABA vs Control, Restricted and Exercise; * *p* < 0.05 Exercise vs Control. Correlation between (**B**) Correlation between Arc expression and body weight in Control, Restricted, Exercise, and ABA rats. (**C**) Representative images of Arc-like immunoreactive elements in the prefrontal cortex. (A1–A4) Row A shows the regional distribution of Arc-like immunoreactive elements in representative prefrontal cortices of (A1) Control, (A2) Restricted, (A3) Exercise, and (A4) ABA rats. Columns B and C and show higher magnifications of Arc immunoreactivity in the (B) prelimbic cortex and (C) infralimbic cortex in the regions of interest (rectangles) represented in column A. Scale bars: 300 µm; 50 µm.

**Figure 10:**
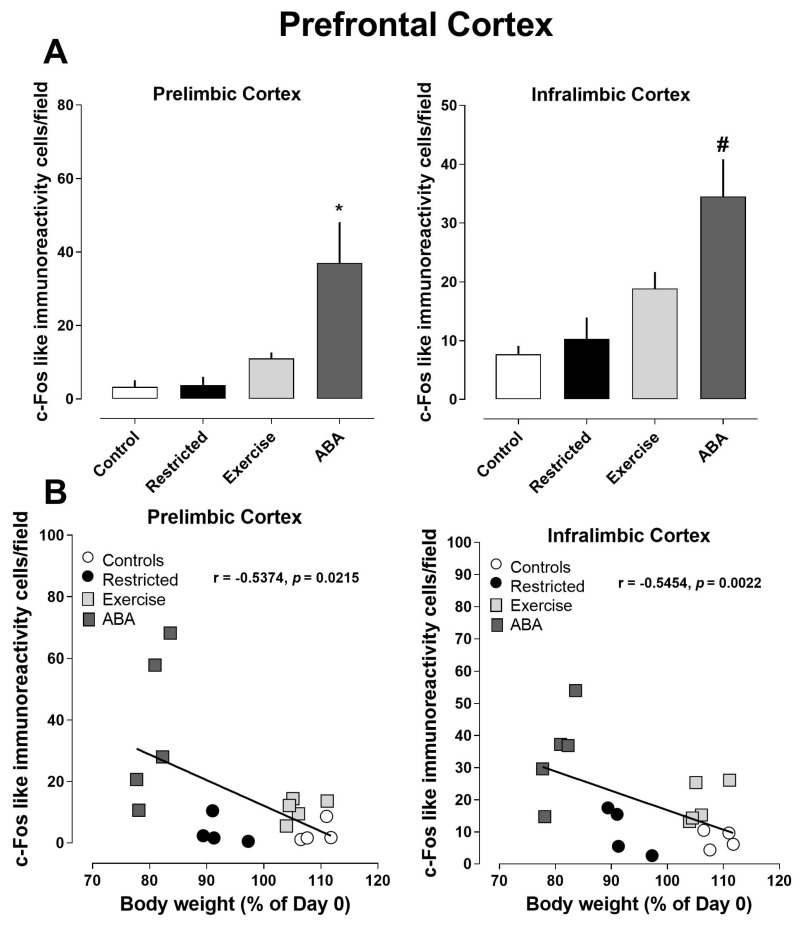
Expression of c-Fos in the prefrontal cortex, and its correlation with body weight (%): (**A**) Stacked bar charts show the counts of c-Fos-positive nuclei/field in the prefrontal cortex. Values represent the mean ± SEM of positive nuclei/field (one-way ANOVA followed by Tukey’s post hoc test). Prelimbic cortex: * *p* < 0.05 vs. Control, Restricted, and Exercise; infralimbic cortex: # *p* < 0.01 vs. Control and Restricted. (**B**) Correlation between c-Fos and body weight in Control, Restricted, Exercise, and ABA rats.

Significantly increased density of Arc-positive cells was found in both the prelimbic and infralimbic subregions of the prefrontal cortex in the ABA and Exercise groups as compared to the Control and Restricted groups (one-way ANOVA: prelimbic cortex, F (3, 28) = 12.16, *p* = 0.0082, *p* < 0.0001; infralimbic cortex, F (3, 28) = 19.68, *p* < 0.0001) (Figure 9A,B). Pearson’s correlations revealed that increased density of Arc-positive cells was negatively correlated with body weight (prelimbic cortex r = −0.5232, *p* = 0.0021; infralimbic cortex r = −0.6345, *p* < 0.0001) (Figure 9B). Regarding c-Fos, immunoreactivity was found to be increased only in the ABA group (one-way ANOVA: prelimbic cortex, F (3, 14) = 12.16, *p* = 0.0060; infralimbic cortex, F (3, 14) = 8.166, *p* = 0.0022) (Figure 10A). Also, in this case, the increase in c-Fos was negatively correlated with the decrease in body weight (prelimbic cortex r = −0.5374, *p* = 0.0215; infralimbic cortex r = −0.5454, *p* = 0.0192) (Figure 10B).

## 4. Discussion

In this study we evaluated the levels of Arc and c-Fos, used as markers for neural plasticity and neural activation respectively, in rats subjected to the ABA model, a validated animal model of AN. We focused our analyses specifically in mesocorticolimbic brain areas such as the hippocampus, the nucleus accumbens and the prefrontal cortex, whose structures and functionalities have been found impaired in AN [21]. In agreement with our previous studies, the ABA group, who was on restricted feeding schedule together with free access to a running wheel, showed a substantial decrease in body weight [17,18]. Contrarily, Restricted group, who was subjected to the same feeding schedules imposed to ABA group, showed a marginal body weight loss, while Control and Exercise groups, who had unlimited access to food, continued to gain body weight reflecting the normal growth of female Sprague Dawley rats during time [17]. Moreover, ABA rats showed a steady increase in RWA and ran more than animals with free access to food, confirming that food restriction leads animals toward a compulsive exercise on the activity wheel. As expected, in Exercise rats the increase in RWA is lower when compared to the ABA group [17]. 

At the molecular level, our immunochemistry data clearly showed an alteration in the density of Arc-positive cells in the ABA group. More specifically, Arc-positive cells were significantly increased in both hippocampal subdivisions (dorsal and ventral) and their subregions (CA1, CA3, and dentate gyrus) when compared to the other experimental groups (Control, Restricted, and Exercise groups). The increase in the number of Arc-positive cells was also found in all subregions of the nucleus accumbens shell (dorsomedial, ventromedial, and ventrolateral) and in the nucleus accumbens core, as well as in the prelimbic and infralimbic subareas of the prefrontal cortex.

It is well established that Arc is expressed at low levels but is rapidly induced by neuronal activity [22]. To assess neuronal activity in our animals, we performed immunohistochemistry for the activity marker c-Fos. Overall, ABA rats showed higher c-Fos activation levels compared to the other experimental groups, highlighting that exposure to ABA conditions leads to neuronal activation in almost all of the brain areas analyzed. More specifically, the number of c-Fos-positive cells was significantly increased in the dentate gyrus of the dorsal hippocampus, as well as in all subregions (CA1, CA3, and dentate gyrus) of the ventral hippocampus. Moreover, c-Fos-positive cells were also increased in the nucleus accumbens shell (dorsomedial, ventromedial, and ventrolateral) and core. Lastly, c-Fos-positive cells were increased in both subareas of the prefrontal cortex. Our data on c-Fos are in line with previous studies reporting neuronal activation in different brain areas of ABA rats [23]. On the other hand, Milton et al. showed that chemogenetic suppression of the medial prefrontal cortex- nucleus accumbens shell pathway was able to attenuate body weight loss in rats exposed to ABA conditions [24]. More specifically, rats keep body weight above 80% of baseline compared to control rats, and this was mainly driven by an increase in food intake. Conversely, chemogenetic stimulation of this pathway exacerbated food-restriction-evoked hyperactivity [24]. Consistent with this, brain imaging techniques indicate altered neural activation in several limbic regions of anorexic patients, which seems to contribute to the development and/or the maintenance of AN [25,26]. It should be noted that the changes in c-Fos observed in our ABA animals broadly resembled the changes in Arc, except for the CA1 and CA3 subregions of the dorsal hippocampus, where the alterations of this marker were not statistically significant compared to the other experimental groups.

As already mentioned, Arc plays an important role in various forms of synaptic plasticity, including long-term potentiation (LTP), long-term depression (LTD), and homeostatic plasticity [27,28,29]. Multiple lines of evidence suggest that a dysregulation of Arc expression may be implicated in the abnormal synaptic plasticity associated with neuropsychiatric disorders [30]. As already mentioned, the ABA model has provided consistent evidence for neuroplasticity changes in specific brain areas of the mesocorticolimbic circuit, which seem to strongly influence the maintenance of the anorexic phenotype. For example, ABA induction strongly alters the morphology of CA1 pyramidal neurons, with reduced dendritic length and decreased branching in the dorsal hippocampus, while dendritic branching increases in the ventral hippocampus [7,8]. Also, ABA vulnerability positively correlated with the levels of NR2A- and NR2B-NMDA receptors on pyramidal neurons in the hippocampus [9]. Additionally, ketamine, as an NMDA receptor antagonist, can reduce ABA’s maladaptive behaviors through changes in the prevalence of NR2B-containing NMDA receptors at excitatory synapses in the medial prefrontal cortex [31]. On the other hand, Mottarlini and collaborators found that the induction of the ABA phenotype is linked with a reorganization of the glutamatergic synapse in the nucleus accumbens [10]. More specifically, they found a switch in the AMPA and NMDA subunit composition with increased GluA1/A2 ratio and GluN2A/2B ratio, respectively. The same authors also found that ABA induction altered the composition and structure of the glutamatergic synapse in the prefrontal cortex, with reduced levels of GluN1 NMDA and GluA2 AMPA receptor subunits, as well as reduced dendritic spine density [11]. It is well established that AMPA and NMDA receptors with different synaptic locations and subunit compositions are also required for the induction and expression of various forms of synaptic plasticity, including LTP and LTD [32]. Moreover, the number and subunit composition of synaptic AMPA and NMDA receptors on synapses change dynamically in response to neuronal activity [33,34]. It is important to highlight that Arc is related to multiple forms of glutamatergic plasticity and it selectively modulates the trafficking of AMPA receptors [35]. Furthermore, neuronal activation mediated by metabotropic and ionotropic glutamate receptors plays central roles in the regulation of Arc expression [36,37]. For example, it has been demonstrated that pharmacological or synaptic activation of NMDA receptors increases Arc protein levels, while their blockage reduces the expression of Arc [36]. Thus, the Arc changes in our ABA animals could be related to a bidirectional relationship between Arc and glutamatergic synapse plasticity and neurotransmission. In addition to neuronal activity, Arc expression in the brain is also influenced by several neurotransmitters other than glutamate. For example, elevation of brain serotonin induced Arc expression particularly in the cortical and striatal areas [38]. Also, the activation of dopamine D1 receptors increased Arc gene expression in the corticostriatal brain regions of adult rats [39]. In our recent study, we demonstrated that ABA induction was associated with significant changes in the corticolimbic contents of dopamine and serotonin [40]. More specifically, we found that dopamine levels significantly increased in the cortex, prefrontal cortex, and nucleus accumbens, while serotonin was significantly enhanced in the nucleus accumbens and hippocampus of ABA rats. Consistent with this, it is possible that the increase in Arc-positive cells observed in our animals could also be related to the changes in dopamine and serotonin levels.

In ABA rats, Arc and c-Fos changes were negatively correlated with body weight loss, which in this group was due to the combination of time-limited food availability and running-wheel activity. It has been demonstrated that food restriction changes the surface expression of GluA1-containing AMPA receptors in the nucleus accumbens [41]. On the other hand, Garcia and collaborators showed the protective effect of exercise in an animal model of Parkinson’s disease by promoting the increase in Arc expression with subsequent changes on AMPA receptors in the motor cortex [42]. Importantly, exercise training during childhood–adolescence reversed the impairment of learning and memory in prenatally di-(2-ethylhexyl)-phthalate-exposed rats by increasing the expressions of hippocampal BDNF, NR1, and Arc [43]. Our study indicated that alterations in both Arc and c-Fos were not specific to the ABA group. The Exercise group also showed a significant increase in Arc in the nucleus accumbens core, as well as in the prelimbic and infralimbic subareas of the prefrontal cortex, when compared with the Control group. Moreover, the Exercise group had significantly increased c-Fos in the CA1 subregion of the ventral hippocampus. When looking at the Restricted group, the feeding schedule provided without exercise induced a significant increase in Arc in the dorsomedial shell. Thus, in some of the analyzed brain areas, the two variables manipulated in the ABA model were able to alter the levels of Arc and c-Fos when applied separately. It should be noted that, in these two experimental groups, a trend towards an increase in the two markers was also observed in other analyzed brain areas. Therefore, significant changes in Arc expression in ABA rats could also be related to both food restriction and physical hyperactivity, which might act synergistically.

## 5. Conclusions

To the best of our knowledge, this is the first study showing an alteration in Arc expression in rats subjected to the ABA model, in specific brain areas of the mesocorticolimbic circuit that are important for a multitude of functions relevant to AN, including reward, emotion, motivation, and cognition, as well as homeostatic and hedonic aspects of feeding behavior [6]. However, the ABA model presents some limitations, as it cannot reproduce some traits of the complex behavioral features of human AN patients, and data obtained from animals must be taken with caution [5]. Although further studies are needed to further elucidate the role of Arc in AN, our data indicate that it could contribute to ABA’s maladaptive behaviors, and its pharmacological manipulation may provide promising therapeutic approaches for the treatment of this disorder.

## Figures and Tables

**Table 1:** Behavioral parameters during the ABA induction phase.

	Group	BL	Day1	Day 2	Day 3	Day 4	Day 5	Day 6
**Body weight** **(% of BL)**	Control Restricted Exercise ABA	100 100 100 100	103.3 ± 0.6 95.9 ± 0.5 ^$^ 102.3 ± 0.5 93.0 ± 0.5 *^#§^	104.3 ± 0.8 95.6 ± 0.9 ^$^ 103.6 ± 0.5 **89.5 ± 0.6 ^#§^**	105.3 ± 1 93.8 ± 0.5 ^$^ 104.2 ± 0.6 **85.9 ± 1.1 ^#§^**	105.8 ± 0.9 93.8 ± 0.7 ^$^ 105.0 ± 0.6 **83.4 ± 1.2 ^#§^**	108.2 ± 1 93.6 ± 0.9 ^$^ 106.6 ± 0.9 **80.9 ± 0.9 ^#§^**	109.7 ± 0.8 92.9 ± 1 ^$^ 109.2 ± 0.9 **79.1 ± 1 ^#§^**
**Food intake** **(g/24 h)**	Control Restricted Exercise ABA	17.2 ± 0.6 16.0 ± 0.5 17.4 ± 0.9 15.7 ± 0.6	16.1 ± 0.7 7.0 ± 0.5 ^$^ 16.8 ± 0.5 4.9 ± 0.3 *^§^	16.0 ± 0.6 7.9 ± 0.7 ^$^ 17.1 ± 1 **5.9 ± 0.3 ^§^**	16.0 ± 0.7 8.5 ± 0.4 ^$^ 16.9 ± 0.7 **7.2 ± 0.2 ^§^**	16.8 ± 0.5 8.6 ± 0.6 ^$^ 19.2 ± 0.9 **8.3 ± 0.5 ^§^**	17.4 ± 1 8.1 ± 0.5 ^$^ 19.2 ± 0.9 **8.6 ± 0.3 ^§^**	- - - -
**RWA**	Exercise ABA	13,023 ± 2838 13,565 ± 1850	15,428 ± 3029 15,793 ± 2546	14,814 ± 3143 **24,563 ± 3631 ^§^**	16,519 ± 3450 **26,047 ± 2980 ^§^**	14,889 ± 1666 **25,212 ± 1682 ^§^**	18,244 ± 2464 **30,851 ± 2294 ^§^**	17,402 ± 1609 **33,472 ± 3193 ^§^**

Measures of body weight (% of baseline, BL), food intake (g/24 h), and running-wheel activity (RWA) in the Control, Exercise, Restricted, and ABA groups during the 6 days of the ABA induction phase. Data are presented as the mean ± SEM (*n* = 8 rats per group). Statistical analysis was performed by two-way ANOVA, followed by Bonferroni’s post hoc test. Body weight: § *p* < 0.001 ABA vs. Control and Exercise; * *p* < 0.05, # *p* < 0.001 ABA vs. Restricted; $ *p* < 0.001 Restricted vs. Control and Exercise. Food intake: § *p* < 0.001 ABA vs. Control and Exercise; * *p* < 0.05 ABA vs. Restricted; $ *p* < 0.001 Restricted vs. Control and Exercise. RWA: § *p* < 0.001 ABA vs. Exercise. Significant differences are highlighted in bold in the table.

## Data Availability

The data are available upon request from the corresponding author.

## References

[1] van Eeden A.E., van Hoeken D., Hoek H.W. (2021). Incidence, prevalence and mortality of anorexia nervosa and bulimia nervosa. Curr. Opin. Psychiatry.

[2] American Psychiatric Association (APA) (2013). Diagnostic and Statistical Manual of Mental Disorders.

[3] Dalle Grave R., Calugi S., Marchesini G. (2008). Compulsive exercise to control shape or weight in eating disorders: Prevalence, associated features, and treatment outcome. Compr. Psychiatry.

[4] Himmerich H., Hotopf M., Shetty H., Schmidt U., Treasure J., Hayes R.D., Stewart R., Chang C.K. (2019). Psychiatric comorbidity as a risk factor for mortality in people with anorexia nervosa. Eur. Arch. Psychiatry Clin. Neurosci..

[5] Spadini S., Ferro M., Lamanna J., Malgaroli A. (2021). Activity-based anorexia animal model: A review of the main neurobiological findings. J. Eat. Disord..

[6] Frank G.K., Shott M.E., DeGuzman M.C. (2019). Recent advances in understanding anorexia nervosa. F1000Research.

[7] Chowdhury T.G., Barbarich-Marsteller N.C., Chan T.E., Aoki C. (2014). Activity-based anorexia has differential effects on apical dendritic branching in dorsal and ventral hippocampal CA1. Brain Struct. Funct..

[8] Chowdhury T.G., Ríos M.B., Chan T.E., Cassataro D.S., Barbarich-Marsteller N.C., Aoki C. (2014). Activity-based anorexia during adolescence disrupts normal development of the CA1 pyramidal cells in the ventral hippocampus of female rats. Hippocampus.

[9] Chen Y.W., Actor-Engel H., Sherpa A.D., Klingensmith L., Chowdhury T., Aoki C. (2017). NR2A- and NR2B-NMDA receptors and drebrin within postsynaptic spines of the hippocampus correlate with hunger-evoked exercise. Brain Struct. Funct..

[10] Mottarlini F., Bottan G., Tarenzi B., Colciago A., Fumagalli F., Caffino L. (2020). Activity-Based Anorexia Dynamically Dysregulates the Glutamatergic Synapse in the Nucleus Accumbens of Female Adolescent Rats. Nutrients.

[11] Mottarlini F., Targa G., Bottan G., Tarenzi B., Fumagalli F., Caffino L. (2022). Cortical reorganization of the glutamate synapse in the activity-based anorexia rat model: Impact on cognition. J. Neurochem..

[12] Ho E.V., Klenotich S.J., McMurray M.S., Dulawa S.C. (2016). Activity-Based Anorexia Alters the Expression of BDNF Transcripts in the Mesocorticolimbic Reward Circuit. PLoS ONE.

[13] Mottarlini F., Rizzi B., Targa G., Fumagalli F., Caffino L. (2022). Long-lasting BDNF signaling alterations in the amygdala of adolescent female rats exposed to the activity-based anorexia model. Front. Behav. Neurosci..

[14] Shepherd J.D., Bear M.F. (2011). New views of Arc, a master regulator of synaptic plasticity. Nat. Neurosci..

[15] Farris S., Lewandowski G., Cox C.D., Steward O. (2014). Selective localization of arc mRNA in dendrites involves activity- and translation-dependent mRNA degradation. J. Neurosci..

[16] El-Sayed M., Hofman-Bang J., Mikkelsen J.D. (2011). Effect of brain-derived neurotrophic factor on activity-regulated cytoskeleton-associated protein gene expression in primary frontal cortical neurons. Comparison with NMDA and AMPA. Eur. J. Pharmacol..

[17] Scherma M., Satta V., Collu R., Boi M.F., Usai P., Fratta W., Fadda P. (2017). Cannabinoid CB1/CB2 receptor agonists attenuate hyperactivity and body weight loss in a rat model of activity-based anorexia. Br. J. Pharmacol..

[18] Collu R., Post J.M., Scherma M., Giunti E., Fratta W., Lutz B., Fadda P., Bindila L. (2020). Altered brain levels of arachidonic acid-derived inflammatory eicosanoids in a rodent model of anorexia nervosa. Biochem. Biophys. Acta Mol. Cell Biol. Lipids.

[19] Paxinos G., Watson C. (2006). The Rat Brain in Stereotaxic Coordinates: Hard Cover Edition.

[20] Pisanu A., Lecca D., Valentini V., Bahi A., Dreyer J.L., Cacciapaglia F., Scifo A., Piras G., Cadoni C., Di Chiara G. (2015). Impairment of acquisition of intravenous cocaine self-administration by RNA-interference of dopamine D1-receptors in the nucleus accumbens shell. Neuropharmacology.

[21] Tadayonnejad R., Majid D.A., Tsolaki E., Rane R., Wang H., Moody T.D., Pauli W.M., Pouratian N., Bari A.A., Murray S.B. (2022). Mesolimbic Neurobehavioral Mechanisms of Reward Motivation in Anorexia Nervosa: A Multimodal Imaging Study. Front. Psychiatry.

[22] Korb E., Finkbeiner S. (2011). Arc in synaptic plasticity: From gene to behavior. Trends Neurosci..

[23] Scharner S., Prinz P., Goebel-Stengel M., Kobelt P., Hofmann T., Rose M., Stengel A. (2016). Activity-Based Anorexia Reduces Body Weight without Inducing a Separate Food Intake Microstructure or Activity Phenotype in Female Rats-Mediation via an Activation of Distinct Brain Nuclei. Front. Neurosci..

[24] Milton L.K., Mirabella P.N., Greaves E., Spanswick D.C., van den Buuse M., Oldfield B.J., Foldi C.J. (2021). Suppression of Corticostriatal Circuit Activity Improves Cognitive Flexibility and Prevents Body Weight Loss in Activity-Based Anorexia in Rats. Biol. Psychiatry.

[25] Lipsman N., Woodside D.B., Lozano A.M. (2015). Neurocircuitry of limbic dysfunction in anorexia nervosa. Cortex.

[26] Eddy K.T., Plessow F., Breithaupt L., Becker K.R., Slattery M., Mancuso C.J., Izquierdo A.M., Van De Water A.L., Kahn D.L., Dreier M.J. (2023). Neural activation of regions involved in food reward and cognitive control in young females with anorexia nervosa and atypical anorexia nervosa versus healthy controls. Transl. Psychiatry.

[27] Minatohara K., Akiyoshi M., Okuno H. (2016). Role of Immediate-Early Genes in Synaptic Plasticity and Neuronal Ensembles Underlying the Memory Trace. Front. Mol. Neurosci..

[28] Peebles C.L., Yoo J., Thwin M.T., Palop J.J., Noebels J.L., Finkbeiner S. (2010). Arc regulates spine morphology and maintains network stability in vivo. Proc. Natl. Acad. Sci. USA.

[29] Wall M.J., Collins D.R., Chery S.L., Allen Z.D., Pastuzyn E.D., George A.J., Nikolova V.D., Moy S.S., Philpot B.D., Shepherd J.D. (2018). The Temporal Dynamics of Arc Expression Regulate Cognitive Flexibility. Neuron.

[30] Molteni R., Calabrese F., Chourbaji S., Brandwein C., Racagni G., Gass P., Riva M.A. (2010). Depression-prone mice with reduced glucocorticoid receptor expression display an altered stress-dependent regulation of brain-derived neurotrophic factor and activity-regulated cytoskeleton-associated protein. J. Psychopharmacol..

[31] Li J., Chen Y.W., Aoki C. (2023). Ketamine ameliorates activity-based anorexia of adolescent female mice through changes in the prevalence of NR2B-containing NMDA receptors at excitatory synapses that are in opposite directions for of pyramidal neurons versus GABA interneurons in medial prefrontal cortex. Res. Sq..

[32] Massey P.V., Johnson B.E., Moult P.R., Auberson Y.P., Brown M.W., Molnar E., Collingridge G.L., Bashir Z.I. (2004). Differential roles of NR2A and NR2B-containing NMDA receptors in cortical long-term potentiation and long-term depression. J. Neurosci..

[33] Shepherd J.D., Huganir R.L. (2007). The cell biology of synaptic plasticity: AMPA receptor trafficking. Ann. Rev. Cell Dev. Biol..

[34] Lau C.G., Zukin R.S. (2007). NMDA receptor trafficking in synaptic plasticity and neuropsychiatric disorders. Nat. Rev. Neurosci..

[35] Chowdhury S., Shepherd J.D., Okuno H., Lyford G., Petralia R.S., Plath N., Kuhl D., Huganir R.L., Worley P.F. (2006). Arc/Arg3.1 interacts with the endocytic machinery to regulate AMPA receptor trafficking. Neuron.

[36] Steward O., Worley P.F. (2001). Selective targeting of newly synthesized Arc mRNA to active synapses requires NMDA receptor activation. Neuron.

[37] Bloomer W.A.C., VanDongen H.M.A., VanDongen A.M.J. (2008). Arc/Arg3.1 translation is controlled by convergent N-methyl-D-aspartate and Gs-coupled receptor signaling pathways. J. Biol. Chem..

[38] Pei Q., Lewis L., Sprakes M.E., Jones E.J., Grahame-Smith D.G., Zetterstrom T.S.C. (2002). Serotonergic regulation of mRNA expression of Arc, an immediate early gene selectively localized at neuronal dendrites. Neuropharmacology.

[39] Fumagalli F., Bedogni F., Frasca A., Di Pasquale L., Racagni G., Riva M.A. (2006). Corticostriatal up-regulation of activity-regulated cytoskeletal-associated protein expression after repeated exposure to cocaine. Mol. Pharmacol..

[40] Giunti E., Collu R., Dedoni S., Castelli M.P., Fratta W., Scherma M., Fadda P. (2023). Food restriction and hyperactivity induce changes in corticolimbic brain dopamine and serotonin levels in female rats. Behav. Brain Res..

[41] Ouyang J., Carcea I., Schiavo J.K., Jones K.T., Rabinowitsch A., Kolaric R., Cabeza de Vaca S., Froemke R.C., Carr K.D. (2017). Food restriction induces synaptic incorporation of calcium-permeable AMPA receptors in nucleus accumbens. Eur. J. Neurosci..

[42] Garcia P.C., Real C.C., Britto L.R. (2017). The Impact of Short and Long-Term Exercise on the Expression of Arc and AMPARs During Evolution of the 6-Hydroxy-Dopamine Animal Model of Parkinson’s Disease. J. Mol. Neurosci..

[43] Sun G.C., Lee Y.J., Lee Y.C., Yu H.F., Wang D.C. (2021). Exercise prevents the impairment of learning and memory in prenatally phthalate-exposed male rats by improving the expression of plasticity-related proteins. Behav. Brain Res..

